# *Isoetes nana*, a new species from the coastal mountains of southeastern Brazil

**DOI:** 10.3897/phytokeys.89.20171

**Published:** 2017-11-03

**Authors:** Jovani B. S. Pereira, Thomas Stützel, Christian Schulz

**Affiliations:** 1 Ruhr-Universität Bochum, Lehrstuhl für Evolution und Biodiversität der Pflanzen, 44780 Bochum, Germany; 2 Vale Institute of Technology, 66055-090, Belém, Pará, Brazil

**Keywords:** Aquatic plant, endemism, lycophytes, taxonomy, spore

## Abstract

*Isoetes nana*, a new species from the coastal mountains of southeastern Brazil (Serra de Itatiaia), is described, illustrated and compared to similar species. This species can be distinguished from similar species by a set of characters that include 5–15 small erect leaves reaching only up to 3.5cm long, megaspores rugulate (rarely laevigate or obscurely cristate) and microspores sparsely echinate. We include a key to identify this new species and spore images for all species that are discussed in this study. *Isoetes nana* is known only from the type locality, where it was reported to occur in small ponds on rocky outcrops at high elevations. We suggest it should be classified as a data deficient species based on the IUCN criteria.

## Introduction

The lycophytes genus *Isoetes* is a heterosporous plant group occurring as aquatic in lakes, temporary ponds, streams, or terrestrial in wet soils (Pfeiffer 1922; Taylor and Hickey 1992). The genus is distributed worldwide and comprises approximately 250 species (Troia et al. 2016). In South America, *Isoetes* is represented by 64 species (Troia et al. 2016). The coastal mountains of southeastern Brazil represent one of the major centres of diversity and endemism in South America (Prado et al. 2015).

*Isoetes* is readily distinguished by its leaves containing a central vascular strand surrounded by four transversely septate lacunae, a single sunken sporangium at the base of the leaves, sporangial trabeculae and a ligule with a basal glossopodium (Gifford and Foster 1987; Pigg 1992; Moran 2004). However, species identification is a complicated task due to the morphological simplicity of the genus that provides few characters and convergence that hides the evolutionary differences amongst the species (Taylor and Hickey 1992). In this context, the most useful characters for species identification are the proportion of the sporangium wall covered by the velum, the sporangial wall colouration, habitat, habit, leaf colour and, especially, the size and ornamentation of the mega- and microspores (Pfeiffer 1922; Taylor and Hickey 2004; Troia and Greuter 2014). The cytology of *Isoetes* (based on *n* = 11) is also particularly important in the taxonomy and correlates strongly with the megaspore size (Kott and Britton 1980; Taylor et al. 1993; Troia 2001; Pereira et al. 2015).

Amongst the main contributions for the taxonomy of *Isoetes* in South America are the studies of Fuchs-Eckert (1982; 1992). However, although the author presented a compilation of species (Fuchs-Eckert 1982; 1992), a large number of these species were never validly published and a comprehensive taxonomic study for these species is still lacking.

Efforts to understand the taxonomy of *Isoetes* prompted us to consult herbarium collections where we noted specimens of an apparently undescribed species from the coastal mountains of southeastern Brazil. Although detected earlier and informally named by Fuchs-Eckert (1982), it was never validly published. Accordingly, in this study, we provide a formal description of the species and a key to identify this new species and similar taxa.

## Material and methods

Fieldwork was carried out by Ernst Heinrich Georg Ule (1854–1915), a German botanist and botanical explorer of the Amazonas and the Brazilian highlands (Stafleu and Cowan 1986), who collected the materials in March 1894 in Serra do Itatiaia, southeastern Brazil. We also tried three times to locate the new species in the field. Specimens from the following herbaria (acronyms following Thiers 2016) were analysed: BHCB, HBR, ICN, PACA, RB, UPCB and VIC (Brazil); NY and US (USA); B, M, HBG, P and K (Europe). Type specimens of all species of *Isoetes* analysed in this study were consulted. The species identification was based on the characters of the leaves and the macro- and microsculpture of the spores.

Scanning electron microscope (SEM) images of the spores were made by transferring the spores to aluminium stubs coated with a carbon adhesive. The stubs were then coated with gold-palladium-alloy in a sputter-coater for 180 s, and then digitally imaged using a Zeiss SIGMA VP. A minimum of 10 megaspores and 20 microspores per species were measured. We used the widely accepted terminology proposed by Punt et al. (2007) for the description of the spores and pollen.

The chromosome numbers of the species here examined were taken from Pereira et al. (2015).

## Taxonomic treatment

### Isoetes nana

Taxon classificationPlantaeIsoetalesIsoetaceae

J.B.S. Pereira sp. nov.

urn:lsid:ipni.org:names:77167028-1

Figs 1A–G ; 7A–C

#### Diagnosis.

*Isoetes nana* can be distinguished from its closely resembling species by a set of characters that include 5–15 small leaves per individual, erect, reaching up to 3.5cm long, megaspore rugulate (rarely laevigate or obscurely cristate) and microspore sparsely echinate.

#### Type.

BRAZIL. Rio de Janeiro: Serra de Itatiaia, March 1894, *Ule 98* (holotype: G!; isotype: HBG!).

#### Description.

Plants terrestrial or aquatic. Corm globose to subglobose, 0.3–0.8cm wide, 2–lobed. Roots conspicuous, dichotomously branched. Leaves 0.6–1.2mm wide at mid length, 1.5–3.5 cm long, 5–15, linear to triangular, straight, erect, apex acute; alae 0.9–1.6 cm long, stretching 1/2–3/4 of total leaf length. Subula semi–terete, olive–green in dry material. Labium present, caducous. Ligule not seen. Velum covering ca. 1/2 of the sporangium surface. Scales absent. Sporangium 1.0–1.5 mm wide, 1.5–2.0 mm long, orbiculate to elliptic, hyaline throughout. Megaspore 480–520 µm diameter (average = 500 µm, *N* = 10), trilete, white, not lustrous, subspheroidal; laesures straight, narrowly triangular, higher than wide, with straight and parallel sides, apex acute, slightly lower close to the pole; macrosculpture of the proximal and distal surfaces rugulate (rarely laevigate or obscurely cristate), microsculpture of the proximal and distal surfaces with terminal ends of anastomosed bars joined forming bacillae or more rarely echinulae; equatorial ridges arched, with straight and parallel sides, rounded. Microspore 29–33µm long (average = 31 µm, *N* = 20), light brown, monolete; laesurae straight, without prominent invagination, macrosculpture on the proximal and distal surface sparsely echinate, echinulae low, microsculpture baculate and granulate.

**Figure 1.**
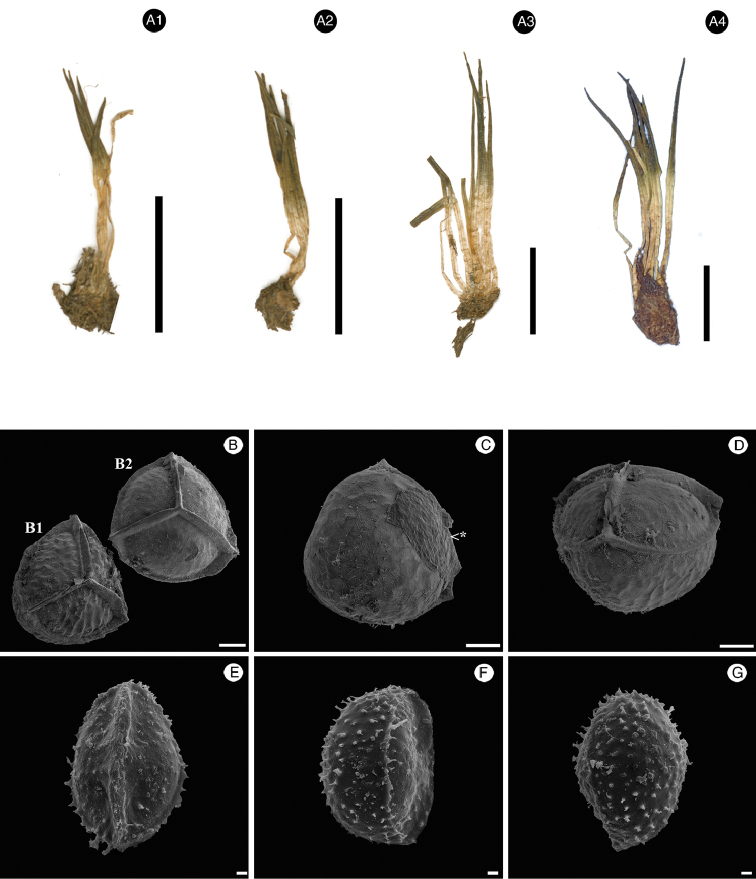
*Isoetes nana* sp. nov. (from the holotype: Ule 98, G). **A1–4** Habit (images are courtesy of the herbarium G) **B–D** Megaspore **B** Proximal view of an obscurely cristate (B1) and rugulate (B2) megaspore **C** Distal view (<*portion of the megaspore covered by sporangium tissue) **D** Equatorial view **E–G** Microspore **E** Proximal view **F** Equatorial view **G** Distal view. Scale bars: **A1–4** = 1 cm; **B–D** = 100 µm; **E–G** = 2 µm.

#### Additional specimens examined.

BRAZIL. Rio de Janeiro: Serra de Itatiaia, em pequenas bacias d’água dos rochedos, Mar. 1894, *Ule s.n.* (P01591972; https://science.mnhn.fr/institution/mnhn/collection/p/item/p01591972).

#### Distribution and habitat.

**Figure 2.**
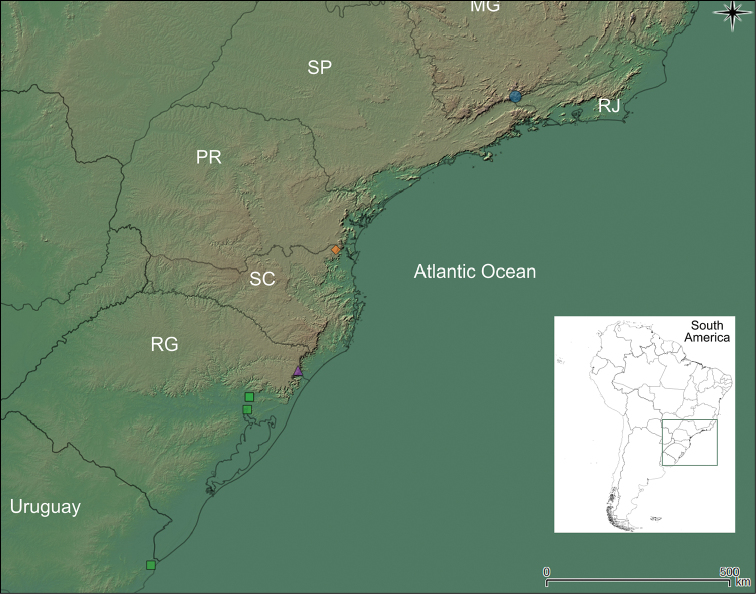
Geographical distribution of *Isoetes nana* (blue circle), *I. maxima* (purple triangle), *I. quiririensis* (orange diamond) and *I. weberi* (green squares) in southeastern South America. In Brazil: MG=Minas Gerais; PR=Paraná; RJ=Rio de Janeiro; RS=Rio Grande do Sul; SC=Santa Catarina; SP=São Paulo.

*Isoetes nana* is known only from the type locality at Serra de Itatiaia, Rio de Janeiro (Figure 2). According to the label on herbarium sheets, the population was found at elevations of about 2300 m and recorded as aquatic, growing in small ponds on rocky outcrops.

#### Comments.

The two collections (Ule 98, kept in G and HBG and Ule *s.n.* kept in P) made by Ule at Serra de Itatiaia (Rio de Janeiro, Brazil) in March 1894 are probably a single collection. Fuchs-Eckert labelled the collection Ule 98 (G) as *Isoetes nana* and also cited this name in a published paper (Fuchs-Eckert 1982: 255). Hickey (1985) also pointed out the unique characters of the megaspore of *I. nana* in his doctoral thesis (Hickey 1985: 142). However, neither Fuchs-Eckert nor Hickey have diagnosed and validly published this new taxon. *Isoetes nana* is known only from these two collections. Although we tried three times to re-collect *Isoetes nana*, without success (all our attempts were with bad weather, including heavy rain), no additional collections have been made until now. The lack of recent collections of this species may probably be due to two main reasons: first, because of its rarity; second, because it has been overlooked (as many *Isoetes* species) by botanists during fieldwork.

**Figure 3.**
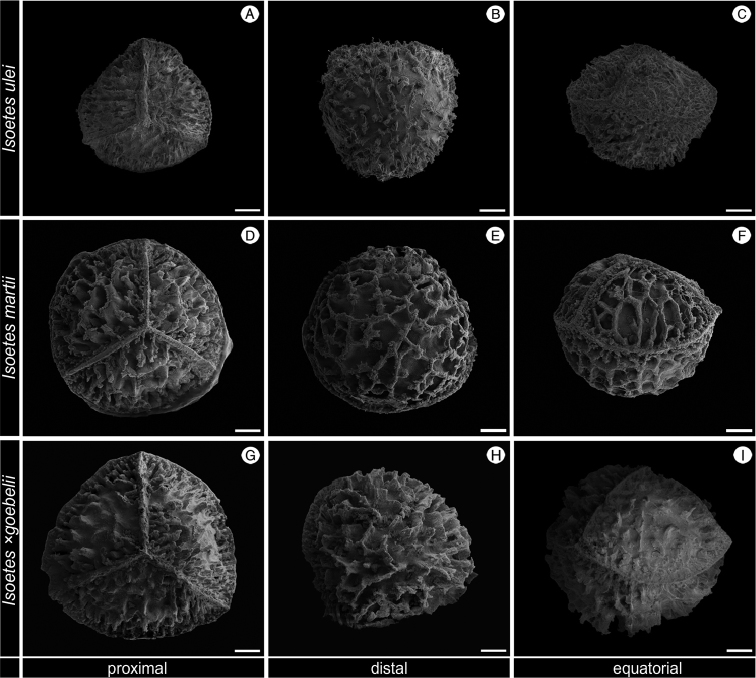
SEM images of the megaspore of *Isoetes ulei* (Ule 3533, HBG), *I. martii* (Pereira 720, UPCB) and *I. × goebelii* (Pereira 718, UPCB). **A–C** Megaspore of *I. ulei* **A** Proximal view **B** Distal view **C** Equatorial view **D–F** Megaspore of *I. martii* **D** Proximal view **E** Distal view **F** Equatorial view **G–I** Megaspore of *I. × goebelii* **G** Proximal view **H** Distal view **I** Equatorial view. All scale bars = 100 µm.

**Figure 4.**
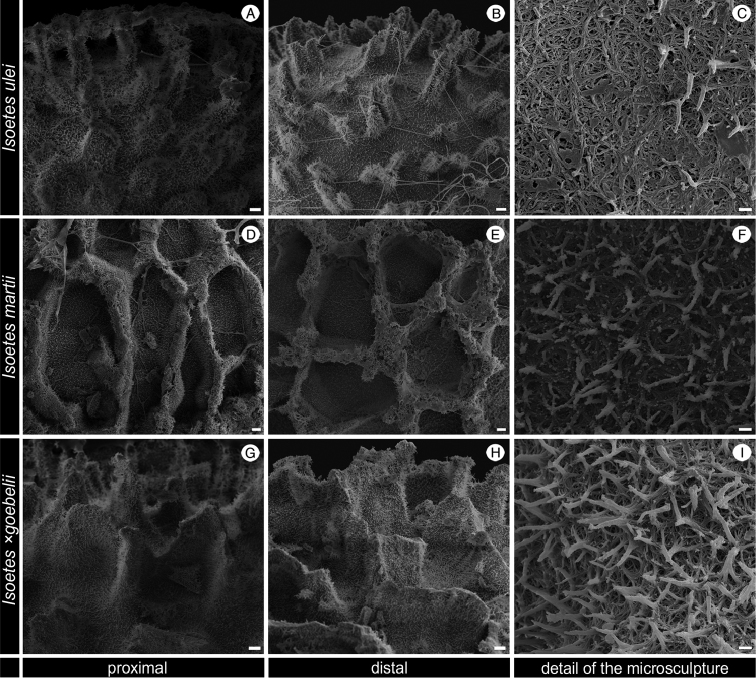
SEM images of the megaspore of *Isoetes ulei* (Ule 3533, HBG), *I. martii* (Pereira 726, UPCB) and *I.* ×g*oebelii* (Pereira 718, UPCB). **A–B** Details of the macrosculpture of the megaspore of *I. ulei* **A** Proximal view **B** Distal view **C** Details of the microsculpture of the megaspore of *I. ulei* in distal view showing the terminal ends of anastomosed bars joined forming echinulae **D–E** Details of the macrosculpture of the megaspore of *martii* **D** Proximal view. E. Distal view **F** Details of the microsculpture of the megaspore of *martii* in distal view showing the terminal ends of anastomosed bars joined forming echinulae **G–H** Details of the macrosculpture of the megaspore of *I. × goebelii* **G** Proximal view **H** Distal view **I** Details of the microsculpture of the megaspore of *I. × goebelii* in distal view showing the terminal ends of anastomosed bars joined forming echinulae. Scale bars: **A, B, D, E, G, H** = 10 µm; **C, F, I** = 1 µm.

**Figure 5.**
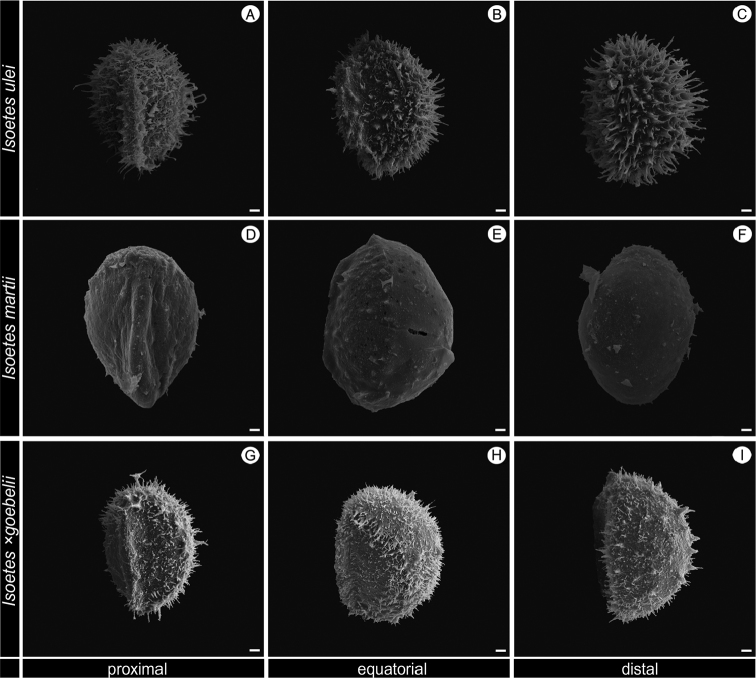
SEM images of the microspore of *Isoetes ulei* (Ule 3533, HBG), *I. martii* (Regnel 1505, B) and *I. × goebelii* (Pereira 718, UPCB). **A–C** Microspore of *I. ulei* **A** Proximal view **B** Distal view **C** Equatorial view **D–F** Microspore of *I. martii* **D** Proximal view **E** Equatorial view **F** Distal view **G–I** Microspore of *I. × goebelii* **G** Proximal view **H** Equatorial view **I** Equatorial view. All scale bars = 2 µm

In the same Serra do Itatiaia, three other *Isoetes* taxa occur: *I. ulei* U. Weber, *I. martii* A. Braun and I. × goebelii U. Weber (*pro sp.*) (*I. martii × Isoetes sp.*) (Figs 3–5). *Isoetes nana* can be easily distinguished from these species and the hybrid by its few leaves (5–15 per individual vs. more than 15 per individual, rarely 10 leaves in *I. martii*) that are short (1.5–3.5 cm vs. > 6 cm) and by its megaspore rugulate, rarely laevigate or obscurely cristate (vs. reticulate or distinctly cristate) and microspore sparsely echinate (vs. laevigate or densely echinate) (Table 1–2).

**Table 1.** Comparison of morphological characters of the *Isoetes* species analysed in this study.

**Taxon**		***Isoetes × goebelii***	***Isoetes martii***	***Isoetes maxima***	***Isoetes nana***	***Isoetes quiririensis***	***Isoetes ulei***	***Isoetes weberi***
Leaf	Number	60–80	10–65	23–95	5–15	25–60	20–40	11–53
	Width (mm)^†^	1.5–1.8	0.8–1.9	0.8–2.2	0.6–1.2	1.8–2.3	1.3–2	0.7–1.4
	Length (cm)	21–30	6–32	15–42	1.5–3.5	25–41	33–60	9–26
	Form	linear	linear	linear to triangular	linear to triangular	linear	linear	linear to filiform
	Position	erect to ascending	erect	erect to ascending	erect	erect	erect	erect to ascending
Alae	Length (cm)	7–7.5	2–10	2.5–14	0.9–1.6	7.5–15	4–8.5	6–12
	Alae/leaf length ratio	1/4–1/3	1/5–2/5	1/5–1/2	1/2–3/4	1/3–2/5	1/10–1/5	1/4–3/4
Ligule	Colour^‡^	dark brown	brown	dark brown	not seen	brown	hyaline	brown
	Form	base cordate and apex attenuate	base cordate and apex attenuate	base cordate and apex acuminate	not seen	base cordate and apex acute	base cordate and apex acute to attenuate	base cordate (apex not seen)
Velum covering		ca. 1/3	1/2–3/4	1/3–3/4	ca. 1/2	3/4 to complete	1/2–3/4	ca. 1/2
Sporangium	Width (mm)	3.5–4	2.3–3.5	3.5–6	1–1.5	2.5–4	2.3–4.2	2.5–4
	Length (mm)	8–9	2.3–7	5–12	1.5–2	5–9	4–6.5	3.5–6
	Colour	hyaline to light brown	hyaline to brown	hyaline to light brown	hyaline	hyaline to light brown	hyaline	hyaline with brown dots
Habitat		aquatic submerged, growing in lakes, rivers and streams	aquatic submerged, growing in lakes, rivers, pods, streams and wet soils	aquatic submerged, growing in streams	aquatic, growing in small ponds on rocky outcrops	aquatic submerged, growing in rivers	aquatic submerged, growing in lakes	aquatic submerged and terrestrial, growing in ponds and wet soils

† Leaf wide at mid length; ‡ Ligula color in dry material

**Table 2.** Comparison of characters of mega– and microspores and chromosome number of *Isoetes* species analysed in this study.

**Species**		***Isoetes × goebelii***	***Isoetes martii***	***Isoetes maxima***	***Isoetes nana***	***Isoetes quiririensis***	***Isoetes ulei***	***Isoetes weberi***
Megaspore	Diameter (µm)	535–717; x=637	640–913; x=705	460–670; x=580	480–520; x=500	477–670; x=567	431–635; x=527	360–490; x=442
	Macrosculpture of the proximal surface	cristate	reticulate	laevigate	rugulate, rarely laevigate or obscurely cristate	verrucate	cristate	rugulate or verrucate
	Macrosculpture of the distal surface	cristate or reticulate	reticulate	laevigate to obscurely verrucate	rugulate, rarely laevigate or obscurely cristate	verrucate	cristate	rugulate or verrucate
	Microsculpture of the proximal and distal surface (terminal ends of anastomosed bars)	echinulae	echinulae	baccillae	bacillae or rarely echinulae	echinulae or rarely bacillae	echinulae	echinulae
Microspore	Maximum length (µm)	30–36; x=34	32–41; x=36	23–34; x=29	29–33; x= 31	26–34; x=31	25–32; x=29	27–31; x= 29
	Macrosculpture of the proximal surface	densely echinate	laevigate	densely echinate	sparsely echinate	sparsely echinate	densely echinate	sparsely echinate or laevigate
	Macrosculpture of the distal surface	densely echinate	laevigate	densely echinate	sparsely echinate	sparsely echinate	densely echinate	sparsely to densely echinate
Chromosome number		33	44	22	–	22	–	–

**Figure 6.**
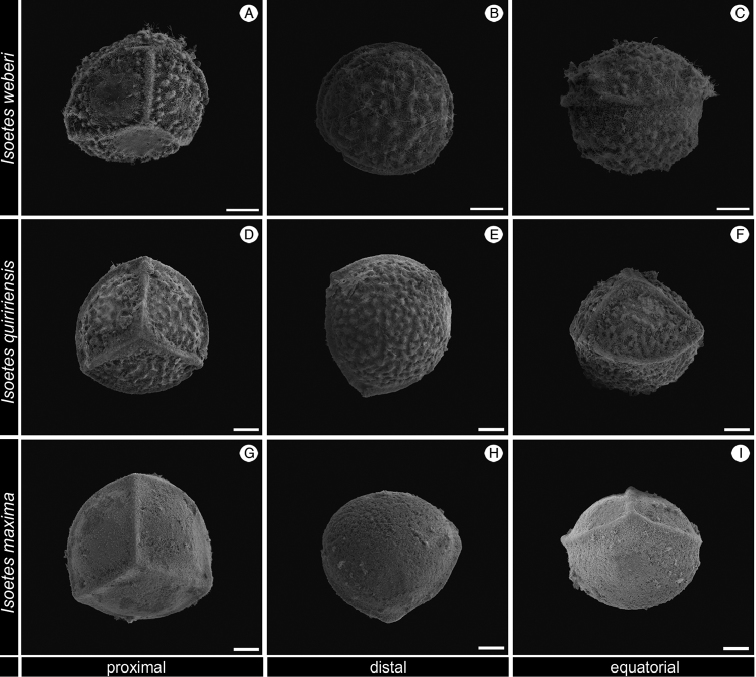
SEM images of the megaspore of *Isoetes weberi* (Herter 95840, US), *I. quiririensis* (Pereira 635, UPCB), *I. maxima* (Pereira 631, UPCB). **A–C** Megaspore of *I. weberi* **A** Proximal view **B** Distal view **C** Equatorial view **D–F** Megaspore of *I. quiririensis* **D** Proximal view **E** Distal view **F** Equatorial view **G–I** Megaspore of *I. maxima* **J** Proximal view **K** Distal view **L** Equatorial view. All scale bars = 100 µm.

**Figure 7.**
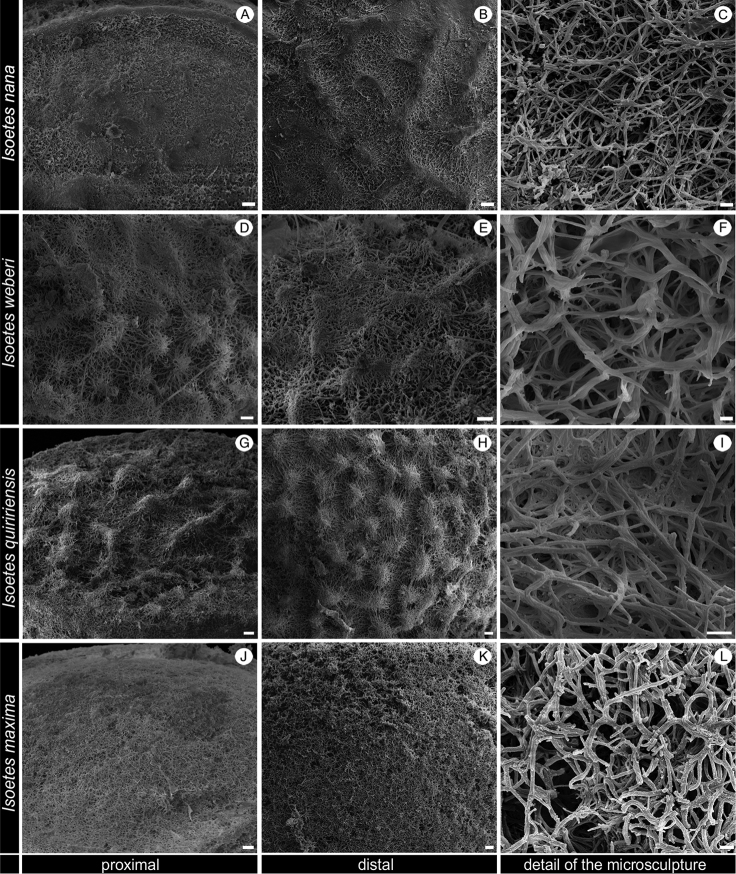
SEM images of the megaspore of *Isoetes nana* (Ule 98, G), *I. weberi* (Herter 95840, US), *I. quiririensis* (Pereira 635, UPCB), *I. maxima* (Pereira 631, UPCB). **A–B** Details of the macrosculpture of the megaspore of *I. nana* **A** Proximal view **B** Distal view **C** Details of the microsculpture of the megaspore of *I. nana* in distal view showing the terminal ends of anastomosed bars joined forming bacillae or more rarely echinulae **D–E** Details of the macrosculpture of the megaspore of *I. weberi* **D** Proximal view **E** Distal view **F** Details of the microsculpture of the megaspore of *I. weberi* in distal view showing the terminal ends of anastomosed bars joined forming echinulae **G–H** Details of the macrosculpture of the megaspore of *I. quiririensis* **G** Proximal view **H** Distal view **I** Details of the microsculpture of the megaspore of *I. quiririensis* in distal view showing the terminal ends of anastomosed bars joined forming echinulae or rarely bacillae **J–K** Details of the macrosculpture of the megaspore of *I. maxima* **J** Proximal view **K** Distal view **L** Details of the microsculpture of the megaspore of *I. maxima* in distal view showing the terminal ends of anastomosed bars joined forming bacillae. Scale bars: **A, B, D, E, G, H, J, K** = 10 µm; **C, F, I, L** = 1 µm.

**Figure 8.**
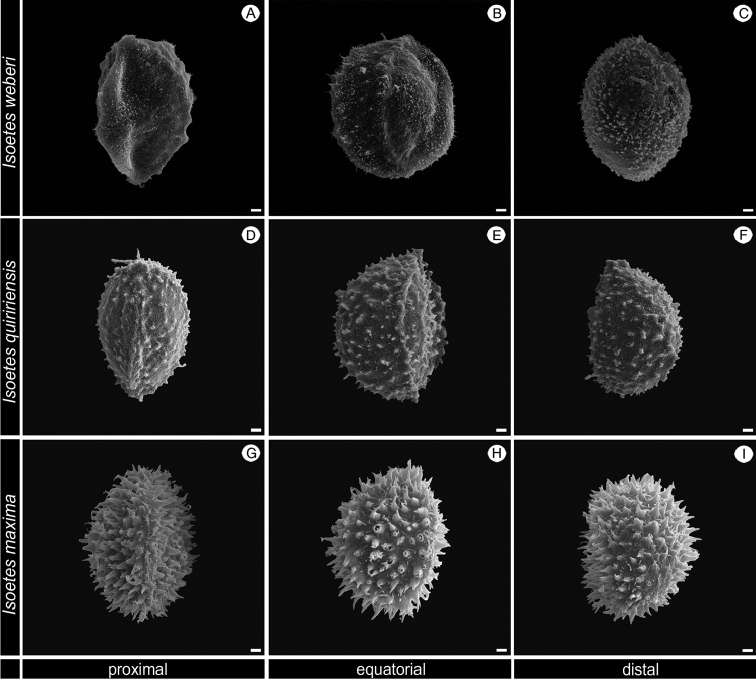
SEM images of the microspore of *Isoetes weberi* (Herter 95840, US), *I. quiririensis* (Pereira 635, UPCB), *I. maxima* (Pereira 631, UPCB). **A–C** Microspore of *I. weberi* **A** Proximal view **B** Equatorial view **C** Distal view **D–F** Microspore of *I. quiririensis* **D** Proximal view **E** Equatorial view **F** Distal view **G–I** Microspore of *I. maxima* **G** Proximal view **H** Equatorial view **I** Distal view. All scale bars = 2 µm.

In the coastal mountains in southern Brazil, three other species with non-reticulate megaspore occur that are similar to this new species (Figure 2; Table 1–2): *I. weberi* U. Weber (Figures 6A–C, 7D–F and 8A–C), *I. quiririensis* J.B.S. Pereira & Labiak (Figures 6D–F, 7G–I and 8D–F) and *I. maxima* Hickey, Macluf & Link-Pérez (Figures 6G–I, 7J–L and 8G–I). *Isoetes nana* can be distinguished by the characters shown in the following taxonomic key and in Tables 1 and 2. Furthermore, although the microsculpture of the megaspore seems to be a reliable source of taxonomic characters and it has been widely used to separate species in *Selaginella* (Schulz et al. 2013; Bauer et al. 2016), the megaspore microsculpture in *Isoetes* is rarely studied and used in the taxonomy (but see Macluf et al. 2003; Troia et al. 2012; Schafran et al. 2016). We observed that the microsculpture of the proximal-distal surfaces of the megaspores of *I. nana* consists of anastomosed bars, whose terminal ends are joined forming bacillae or more rarely echinulae (Figure 7C). Amongst the analysed species, only *I. maxima* shows a similar microsculpture pattern (Figure 7L), while all remaining species present terminal ends of anastomosed bars joined forming echinulae (or more rarely bacillae in *I. quiririensis*) (Figures 4C, F, I and 7F, I). These data show that megaspore microsculpture may provide useful characters not only to identify *I. nana*, but also for the taxonomy of Brazilian *Isoetes*.

Since there is a correlation between spore sizes and ploidy level (Kott and Britton 1980; Troia 2001; Pereira et al. 2015) and *I. nana* presents mega- and microspore sizes that are similar to those of the diploids *I. quiririensis* and *I. maxima* (Table 2), we hypothesise that *I. nana* is also diploid.

#### Conservation status.

Since *I. nana* is currently known from a single (not recently confirmed) locality, it may deserve special attention concerning its conservation status. However, based on our current knowledge on this species and according to IUCN Red List criteria (IUCN 2012), it is assessed here as data deficient (DD).

### Key to taxa of *Isoetes* from Serra do Itatiaia, Rio de Janeiro, Brazil and non-reticulate megaspore species from the coastal mountains of southeastern South America

**Table:** 

1	Megaspores reticulate or cristate	**2**
2	Microspores laevigate on distal surface; megaspores reticulate	***Isoetes martii***
2'	Microspores echinate on distal surface; megaspores cristate (if reticulate, weakly reticulate)	**3**
3	Leaves 21–30 cm long, 60–80 per individual; sporangium 8–9 mm long; megaspores 535–717 µm diam. (average = 637µm), cristate to weakly reticulate; microspores 30–36 µm long (average = 34µm)	***I. × goebelii***
3'	Leaves 33–60 cm long, 20–40 per individual; sporangium 4–6.5mm long; megaspores 431–635 µm diam. (average = 527 µm), clearly cristate; microspores 25–32 µm long (average = 29 µm)	***I. ulei***
1'	Megaspore laevigate, rugulate or verrucate (if cristate, obscurely cristate with small and weakly developed muri)	**4**
4	Leaves up to 3.5 cm long, 5–15 per individual; sporangium up to 1.5 mm wide and up to 2 mm long; laesurae of megaspores narrowly triangular	***I. nana***
4'	Leaves more than 9 cm long, 16-95 per individual (if less than 15 leaves, then greater than 9 cm long); sporangium more than 2.5 mm wide and 3.5 mm long; laesurae of megaspores widely triangular	**5**
5	Sporangia whitish with castaneous spots; megaspores rugulate or verrucate with elongated verrucae (more than 4 times longer than wide), megaspores microsculpture (SEM) with terminal ends of anastomosed bars joined forming echinulae	***I. weberi***
5'	Sporangia whitish or castaneous throughout; megaspores verrucate with rounded to slightly elongated verrucae (not more than twice longer than wide), megaspores microsculpture (SEM) with terminal ends of anastomosed bars joined forming bacillae or echinulae (*I. quiririensis*)	**6**
6	Velum covering 1/3–3/4 of the sporangial surface; megaspore laevigate to obscurely verrucate, megaspores microsculpture (SEM) with terminal ends of anastomosed bars joined forming only bacillae; microspores densely echinate, the spines narrow and sharp	***I. maxima***
6'	Velum covering more than 3/4 of the sporangial surface; megaspores conspicuously verrucate, megaspores microsculpture (SEM) with terminal ends of anastomosed bars joined forming bacillae or more rarely echinulae; microspores sparsely echinate, the spines broad and obtuse	***I. quiririensis***

## Supplementary Material

XML Treatment for Isoetes nana
